# Global Responses of Autopolyploid Sugarcane Badila (*Saccharum officinarum* L.) to Drought Stress Based on Comparative Transcriptome and Metabolome Profiling

**DOI:** 10.3390/ijms24043856

**Published:** 2023-02-14

**Authors:** Shan Yang, Na Chu, Naijie Feng, Bolin Zhou, Hongkai Zhou, Zuhu Deng, Xuefeng Shen, Dianfeng Zheng

**Affiliations:** 1 College of Coastal Agricultural Sciences, Guangdong Ocean University, Zhanjiang 524088, China; 2 National Engineering Research Center for Sugarcane, Fujian Agriculture and Forestry University, Fuzhou 350002, China

**Keywords:** drought, sugarcane, phenylalanine, arginine and proline, transcriptome, metabolome

## Abstract

Sugarcane (*Saccharum* spp. hybrid) is frequently affected by seasonal drought, which causes substantial declines in quality and yield. To understand the drought resistance mechanisms of *S. officinarum*, the main species of modern sugarcane, at a molecular level, we carried out a comparative analysis of transcriptome and metabolome profiling of the sugarcane variety Badila under drought stress (DS). Compared with control group (CG) plants, plants exposed to DS had 13,744 (6663 up-regulated and 7081 down-regulated) differentially expressed genes (DEGs). GO and KEGG analysis showed that the DEGs were enriched in photosynthesis-related pathways and most DEGs had down-regulated expression. Moreover, the chlorophyll content, photosynthesis (Photo), stomatal conductance (Cond), intercellular carbon dioxide concentration (Ci) and transpiration rate (Trmmol) were sharply decreased under DS. These results indicate that DS has a significant negative influence on photosynthesis in sugarcane. Metabolome analysis identified 166 (37 down-regulated and 129 up-regulated) significantly regulated metabolites (SRMs). Over 50% of SRMs were alkaloids, amino acids and their derivatives, and lipids. The five most significantly enriched KEGG pathways among SRMs were Aminoacyl-tRNA biosynthesis, 2-Oxocarboxylic acid metabolism, Biosynthesis of amino acids, Phenylalanine metabolism, and Arginine and proline metabolism (*p* < 0.05). Comparing CG with DS for transcriptome and metabolome profiling (T_CG/DS and M_CG/DS, respectively), we found three of the same KEGG-enriched pathways, namely Biosynthesis of amino acids, Phenylalanine metabolism and Arginine and proline metabolism. The potential importance of Phenylalanine metabolism and Arginine and proline metabolism was further analyzed for response to DS in sugarcane. Seven SRMs (five up-regulated and two down-regulated) and 60 DEGs (17 up-regulated and 43 down-regulated) were enriched in Phenylalanine metabolism under DS, of which *novel.31261*, *Sspon.04G0008060-1A*, *Sspon.04G0008060-2B* and *Sspon.04G0008060-3C* were significantly correlated with 7 SRMs. In Arginine and proline metabolism, eight SRMs (seven up-regulated and one down-regulated) and 63 DEGs (32 up-regulated and 31 down-regulated) were enriched, of which *Sspon.01G0026110-1A* (*OAT*) and *Sspon.03G0002750-3D* (*P5CS*) were strongly associated with proline (r > 0.99). These findings present the dynamic changes and possible molecular mechanisms of Phenylalanine metabolism as well as Arginine and proline metabolism under DS and provide a foundation for future research and sugarcane improvement.

## 1. Introduction

Plants need sufficient water for growth, development and reproduction. As global populations increase and global climate warming accelerates, shortages of fresh water are becoming more frequent and more severe. Drought is a devastating abiotic stress that is expected to affect more than half of the world’s arable land by 2050 [1]. As a result, plants will be increasingly vulnerable to drought stress, resulting in severe yield reduction. Sugarcane is generally grown on sloping land without irrigation, and often suffers from seasonal drought that seriously decreases quality and yield [2]. Increasing sugarcane resistance to drought stress would be the most economical approach to improve sugar productivity. Therefore, understanding the mechanisms of drought tolerance and breeding for drought-resistant sugarcane has been a major goal of plant biologists and sugarcane breeders.

Sugarcane belongs to the genus *Saccharum* and is an important sugar and bioenergy crop that is the source of over 80% of sugar worldwide. The genus *Saccharum* comprises six species: *S. officinarum*, *S. sinense*, *S. bareri*, *S. edule*, *S. robustum*, and *S. spontaneum* [3]. Commercial sugarcane cultivars are generally the result of interspecific hybridization and carry approximately 120 chromosomes, of which 70–80% originate from *S. officinarum*. The remaining 10–20% of chromosomes are from *S. spontaneum*, and a few chromosomes are the product of interspecific recombination [4]. Therefore, *S. officinarum* is the core species in the modern sugarcane varieties and understanding its regulatory mechanisms of drought tolerance at a molecular level is critical for preserving the quality and yield of sugarcane.

Drought stress (DS) results in multiple negative effects on plant morphology as well as physiological and biochemical processes of plants [5]. DS inhibits nutrient uptake, affects gene expression, decreases photosynthesis, and impairs cell elongation and division in plants. These effects are manifested morphologically as yellow leaves, slow growth and decreased yield [6]. To alleviate DS, sugarcane plants mount a variety of physiological and biochemical responses, such as stomatal regulation, osmoregulation, antioxidant enzyme activation, and up/down-regulated gene expression [2]. Advances in RNA-Seq technology have allowed high throughput study of gene expression and transcriptomes with high precision [7]. Transcriptome analysis comparing *Saccharum spontaneum* GX83-10 with normal watering and exposed to DS identified 1325 significantly differentially expressed genes (DEGs) in the DS plants. These genes were enriched in the KEGG pathways of ascorbate and aldarate metabolism, plant hormone signal transduction, and phenylpropanoid biosynthesis [8]. These results indicated that mechanisms of sugarcane drought resistance involve complex metabolic networks. Furthermore, a gene regulatory network for ROC22 and Badila exposed to DS compiled using weighted gene co-expression network analysis (WGCNA) revealed several important candidate genes including the classical transcription factors *NAC87*, *JAMYB*, *bHLH84*, *NAC21/22*, *HOX24* and *MYB102*, and genes related to antioxidants and trehalose [9]. Through genome-wide in silico analysis of genes encoding dehydration responsive element binding (DREBs) transcription factors in a transcriptome analysis of polyploid *S*. *spontaneum*, *SsDREB1F*, *SsDREB1L*, *SsDREB2D*, and *SsDREB2F* were shown to respond positively to DS and to exhibit up-regulated expression [10].

DS disrupts a variety of normal metabolic activities in plants, including enzyme activities, as well as production of osmotic substances and secondary metabolites. The exposure of plants to DS induces differential expression of genes, and these changes are accompanied by significant changes in the levels of several metabolic substances. To maintain essential metabolism and acclimate through a new steady state in response to prevailing stress conditions, metabolic networks must be reconfigured [11]. Metabolomics is an effective tool for gathering comprehensive information on metabolite profiling and to carry out analyses of metabolic networks. For example, in rice, limited water can distinctly affect metabolite accumulation, particularly the products of the phenylpropanoid pathway that is involved in scavenging mechanisms. Moreover, the accumulation of most amino acids was significantly higher in IR64 (drought-susceptible) than in Apo (drought-tolerant) rice cultivars [12]. In sugarcane, an analysis of variations in the metabolomes of sugarcane stalks revealed factors associated with sugarcane bitterness, as evidenced by significant enrichment of DEGs involved in flavone and flavonol biosynthesis pathways in bitter sugarcane compared to sweet sugarcane [13]. Combined transcriptome and metabolome analyses are also often used to reveal gene-metabolite regulatory networks for agronomic traits or responses to specific stresses in plants. Yi et al. used metabolome and transcriptome analyses to show that three cyanidin derivatives (cyanidin 3-O-glucoside, cyanidin 3-O-6″-malonyl-glucoside, and cyanidin O-syringic acid), one delphinidin derivative, and one pelargonidin derivative are the main metabolites that regulate the coloring mechanisms of red longan pericarp [14]. Another study analyzed the transcriptome and metabolome of chlorotic and non-chlorotic sugarcane leaves and found that chlorophyll production, metal ion metabolism, photosynthesis, and some metabolites in the phenylpropanoid biosynthesis pathway were considerably altered in chlorotic ratoon sugarcane leaves [15]. These studies demonstrate that combined -omics analyses are valuable for discovering molecular regulatory networks that are affected by different types of stress.

However, to date, information concerning the regulation network of gene-metabolite responses to DS by sugarcane remains limited. To understand the global response of sugarcane to DS, in this study we carried out comparative transcriptome and metabolome profiling of Badila plants at the jointing stage that had normal watering (control group, CG) or drought stress (DS) for 10 days. Using these analyses, we aimed to reveal the molecular regulatory network of genes and metabolites related to drought responses in sugarcane. Results of these analyses will reveal relevant regulatory genes and metabolites in specific pathways that could be used as a basis to improve sugarcane drought tolerance.

## 2. Results

### 2.1. Phenotypic and Physiological Response to Drought Stress

**Figure 1:**
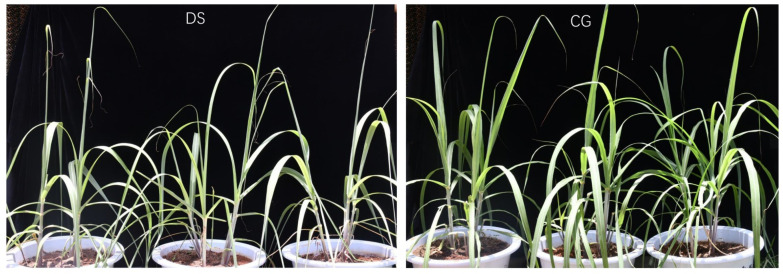
Phenotype of Badila with drought stress (DS; **left**) or normal watering (control group, CG; **right**).

**Figure 2:**
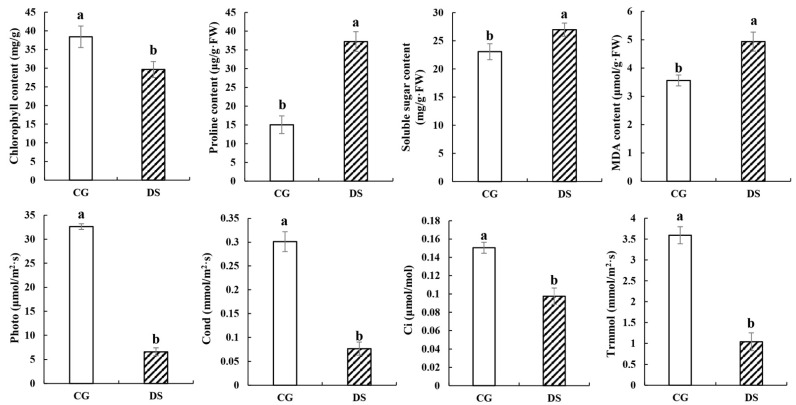
Physiological and biochemical indexes. Photo, Cond, Ci and Trmmol indicate net photosynthesis, stomatal conductance, intercellular carbon dioxide concentration and transpiration rate, respectively. Error bars represent standard deviation among means for three different samples. Different letters on bars indicate statistically significant differences between treatments (*p* < 0.05).

The phenotype of drought stress (DS) group plants exposed to drought stress for 10 days differed significantly from control group (CG) plants that received normal amounts of water. The phenotypic changes included leaf yellowing, leaf rolling and reduced biomass (Figure 1). Compared to CG plants, the chlorophyll content, photosynthesis (Photo), stomatal conductance (Cond), intercellular carbon dioxide concentration (Ci) and transpiration rate (Trmmol) were all markedly decreased under DS. Meanwhile, DS plants had increased proline content, soluble sugar content and MDA content (Figure 2). These changes indicated that sugarcane develops specific phenotypes and physiological responses to drought stress.

### 2.2. Transcriptome Response to DS

**Figure 3:**
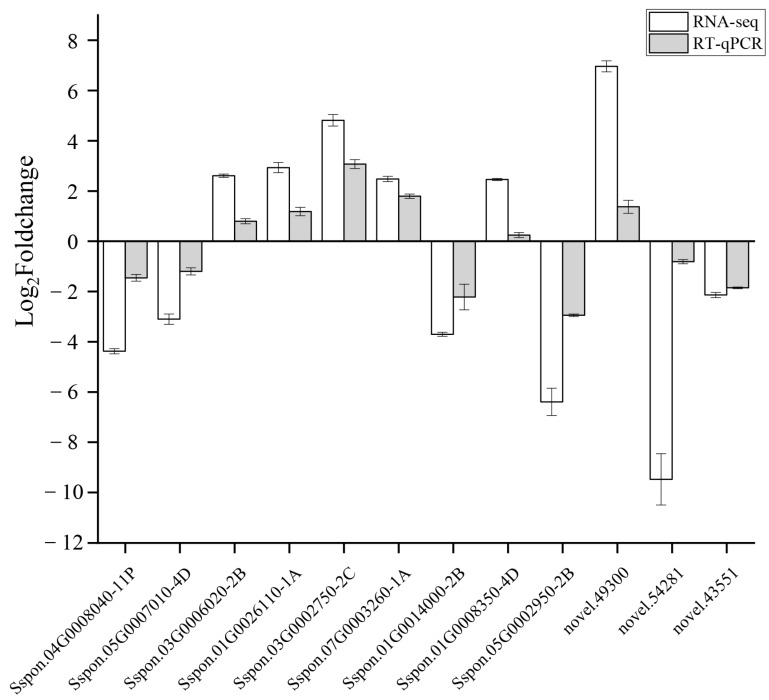
Results of RNA-seq and RT-qPCR for 12 genes. The gene expression level of RNA-seq shows as Log_2_(fold change) and the fold-change is based on the FPKM values of drought stress group relative to control group. The gene expression level of RT-qPCR shows as Log_2_(fold change = 2^−ΔΔCt^).

To obtain clean data for subsequent analysis, raw data of the six transcriptome libraries were filtered by checking sequencing error rates and GC content distribution. The Q20 of the six libraries was above 96.58% and the Q30 was above 90.89%, whereas the GC content was between 51.76% and 56.54%. These results indicate that the accuracy of the experimental data was high and could meet requirements for subsequent bioinformatics analyses (Table S1). Differentially expressed genes (DEGs) were identified using DESeq2 (|log_2_fold change| ≥ 1 and FDR < 0.05). Compared to CG transcripts, 13,744 DEGs were identified, of which 7081 had down-regulated expression and 6663 had up-regulated expression (Figure S1). To validate the accuracy of RNA-seq data, 12 DEGs involved in Phenylalanine metabolism and Arginine and proline metabolism were randomly selected for RT-qPCR analysis (Table S2). The trend of up-regulated and down-regulated expression was consistent between RNA-seq and RT-qPCR for 12 genes, but the absolute value of gene expression level of RT-qPCR was less than those of RNA-seq, especially, for *Sspon.01G0008350-4D* and *novel.54281* (Figure 3). To find out the reason, it may be that the accuracy of RNA-seq technology is different from that of RT-qPCR, or RT-qPCR has more artificial errors (Figure 3). Overall, similar patterns of gene expression were observed between RNA-seq and RT-qPCR data.

**Figure 4:**
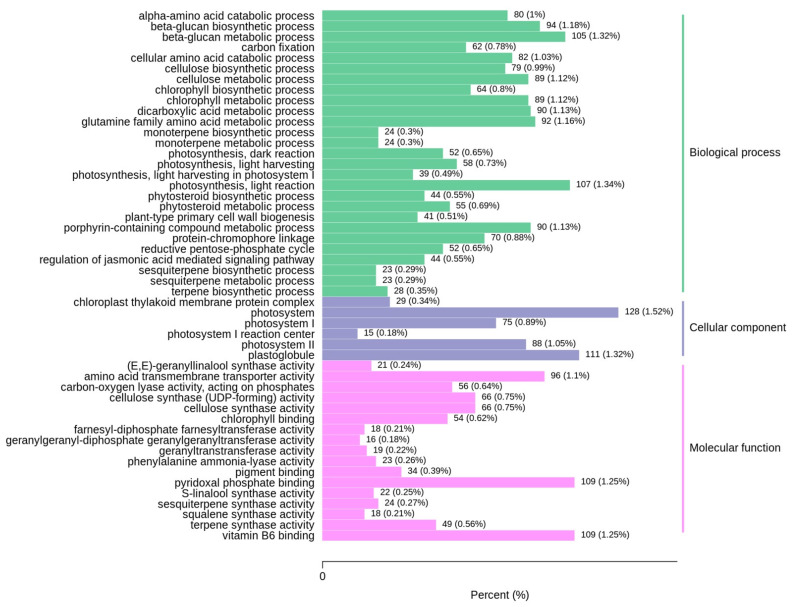
Top 50 enriched GO pathways in a comparison of CG and DS transcriptomes.

GO enrichment analysis showed that 3715 DEGs were enriched in 517 GO terms in a comparison of CG and DS transcriptomes (T_CG/DS (*p* < 0.005)). Of these, 308 GO terms belonged to biological process (BP), 20 belonged to cellular component (CC) and 189 belonged to molecular function (MF) (Table S3). Based on *p*-value, the top-5 enriched terms for BP were carbon fixation, reductive pentose-phosphate cycle, photosynthesis (dark reaction), photosynthesis (light harvesting in photosystem I) and photosynthesis (light harvesting). These pathways contained 62 (19 up-regulated and 43 down-regulated), 52 (14 up-regulated and 38 down-regulated), 52 (14 up-regulated and 38 down-regulated), 39 (39 down-regulated) and 107 (nine up-regulated and 98 down-regulated) DEGs, respectively (Figure 4 and Table S3). Photosystem, photosystem I, plastoglobule, photosystem II and chloroplast thylakoid membrane protein complex were the top five enriched GO terms in CC and contained 128 (four up-regulated and 124 down-regulated), 75 (75 down-regulated), 111 (13 up-regulated and 98 down-regulated), 88 (four up-regulated and 84 down-regulated) and 29 (six up-regulated and 23 down-regulated) DEGs, respectively (Figure 4 and Table S3). For MF, the top five enriched terms were chlorophyll binding, carbon-oxygen lyase activity (acting on phosphates), pigment binding, sesquiterpene synthase activity and cellulose synthase activity. A total of 54 (down-regulated), 56 (two up-regulated and 54 down-regulated), 34 (down-regulated), 24 (one up-regulated and 23 down-regulated) and 66 (five up-regulated and 61 down-regulated) DEGs were identified in these pathways, respectively (Figure 4 and Table S3). Together, these findings showed that the photosynthetic activity of sugarcane had a substantial response to DS and most of the DEGs had down-regulated expression.

**Figure 5:**
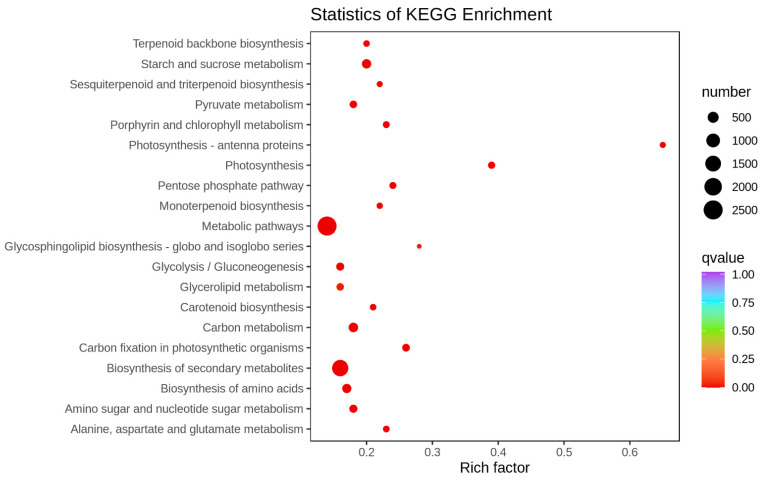
Top 20 enriched KEGG pathways in a comparison of CG and DS transcriptomes.

KEGG pathway enrichment analysis produced similar results to the GO analysis, revealing that 3293 DEGs for T_CG/DS were significantly enriched in 45 KEGG pathways (*p* < 0.05) (Table S4). The top five KEGG enriched pathways for comparison of DS with CG were carbon fixation in photosynthetic organisms, photosynthesis-antenna proteins, photosynthesis, carbon metabolism and starch and sucrose metabolism, and contained 131 (30 up-regulated and 101 down-regulated), 44 (44 down-regulated), 99 (12 up-regulated and 87 down-regulated), 299 (106 up-regulated and 193 down-regulated) and 282 (112 up-regulated and 170 down-regulated) DEGs, respectively (Figure 5 and Table S4). Thus, both GO and KEGG indicated that DS had a significant effect on sugarcane photosynthesis that manifested as down-regulated gene expression.

### 2.3. Metabolome Response to DS

**Figure 6:**
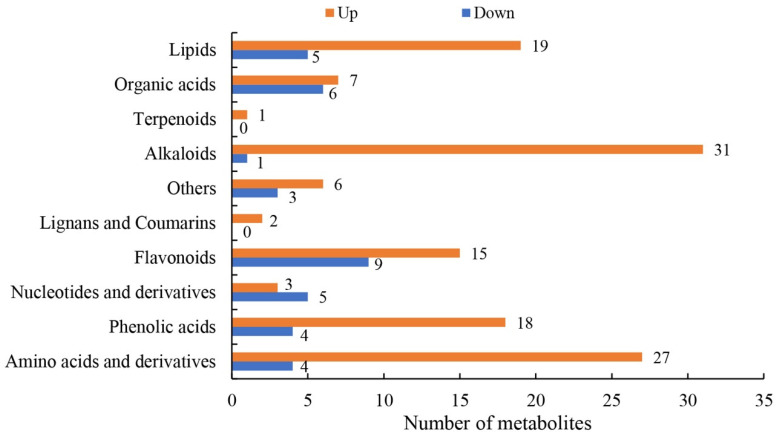
Classification of significantly regulated metabolites (SRMs).

**Figure 7:**
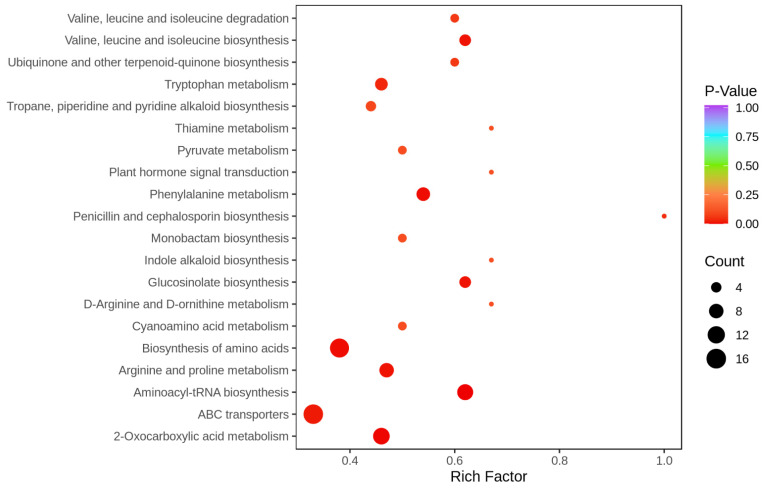
Top 20 enriched KEGG pathways in a comparison of CG and DS metabolomes.

Variations in levels of sugarcane leaf metabolites under different treatment conditions were measured using LC-MS/MS. A heat map analysis of the metabolites showed significant differences between CG and DS plants, indicating that DS affected metabolite production or usage (Figure S2). Compared with CG plants, DS plants had 166 significantly regulated metabolites (SRMs), of which 37 SRMs were down-regulated and 129 SRMs were up-regulated (Figure S3). These SRMs were classified into 10 types, namely lipids, organic acids, terpenoids, alkaloids, others (sugars and alcohols), lignans and coumarins, flavonoids, nucleotides and their derivatives, phenolic acids and amino acids and amino acid derivatives (Figure 6). The alkaloids, amino acids and their derivatives, lipids, flavonoids and phenolic acids contained 32 (31 up-regulated and one down-regulated), 31 (27 up-regulated and four down-regulated), 24 (19 up-regulated and five down-regulated), 24 (15 up-regulated and nine down-regulated) and 22 (18 up-regulated and four down-regulated) SRMs that accounted for 19.28%, 18.67%, 14.46%, 14.46% and 13.25%, respectively, of the total (Figure 6). KEGG analysis showed that the top five significantly enriched KEGG pathways were Aminoacyl-tRNA biosynthesis, 2-Oxocarboxylic acid metabolism, Biosynthesis of amino acids, Phenylalanine metabolism, and Arginine and proline metabolism (*p* < 0.05) that were represented by 10 (up-regulated), 11 (eight up-regulated and three down-regulated), 15 (12 up-regulated and three down-regulated), 7 (five up-regulated and two down-regulated) and eight (seven up-regulated and one down-regulated) SRMs, respectively (Figure 7 and Table S5). Thus, DS appears to significantly affect synthesis and accumulation of metabolites, and levels of most of the SRMs were up-regulated in response to DS.

### 2.4. Combined Transcriptome and Metabolome Analysis

**Figure 8:**
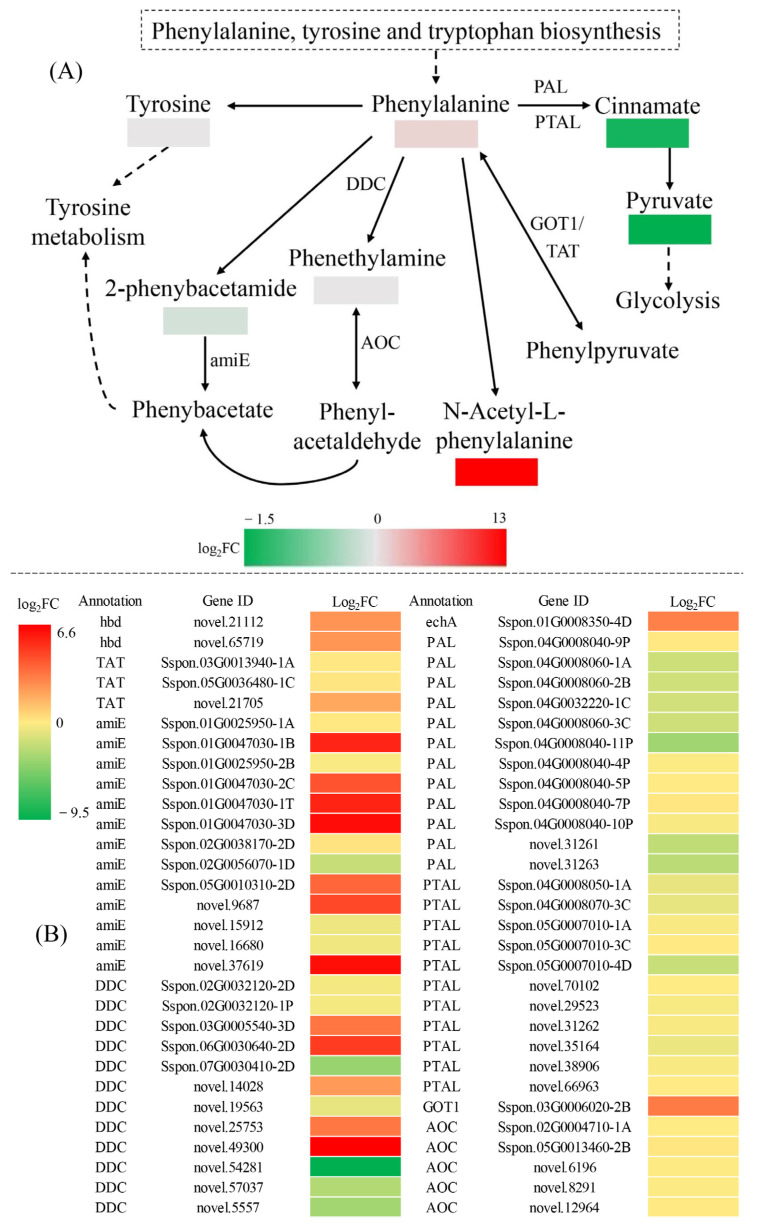
Analysis of DEGs and SRMs in the phenylalanine metabolism pathway. Enriched (**A**) SRMs and (**B**) DEGs color-coded according to fold-change between CG and DS groups. |log_2_FC| denotes |log_2_Fold Change|. Solid line stands for direct reaction. Dashed line stands for indirect reaction, which means it have to go through multiple reactions.

**Figure 9:**
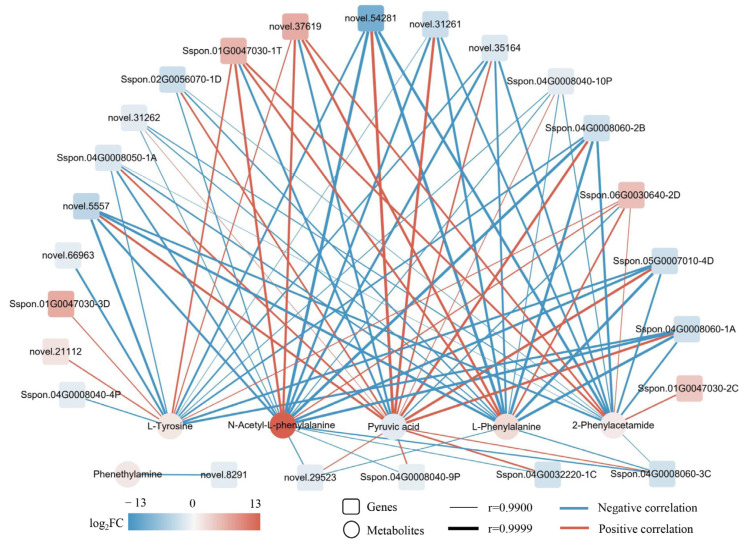
Correlation network of DEGs and SRMs in the phenylalanine metabolism pathway. The genes and metabolites color-coded according to fold-change between the CG and DS groups. |log_2_FC| denotes |log_2_Fold Change|.

To gain insights into gene-metabolite networks that are regulated by DS, we performed a combined transcriptome and metabolome analysis. The resulting Venn diagram showed that Biosynthesis of amino acids, Phenylalanine metabolism and Arginine and proline metabolism were common KEGG-enriched pathways in comparisons of CG and DS transcriptomes (T_CG/DS) and metabolomes (M_CG/DS) (Figure S4, Tables S4 and S5). Phenylalanine metabolism and Arginine and proline metabolism are two branches of amino acid biosynthesis. Compared to CG plants, DS plants had enrichment of seven SRMs (five up-regulated and two down-regulated) and 60 DEGs (17 up-regulated and 43 down-regulated) in the phenylalanine metabolism pathway (Figure 8 and Table S6). DEGs for PAL and PTAL had down-regulated expression, and those for cinnamate and pyruvate were also down-regulated (Figure 8 and Table S6). DEGs associated with phenylalanine, phenethylamine, N-acetyl-L-phenylalanine, 2-phenybacetamide and tyrosine metabolism had up-regulated expression (Figure 8 and Table S6). Taken together, phenylalanine metabolism actively responded to DS, resulting in a reduced content of cinnamate as well as pyruvate, that in turn reduced levels of downstream phenylalanine secondary metabolites and also decreased respiration needed for metabolic activities. A gene-metabolite correlation analysis (r > 0.9 and *p* < 0.01) showed that phenylalanine, cinnamate, phenethylamine, N-acetyl-L-phenylalanine, 2-phenybacetamide, tyrosine and pyruvate were significantly correlated with 43 genes (14 positively and 29 negatively correlated), 35 genes (26 positively and nine negatively correlated), 39 genes (11 positively and 28 negatively correlated), 42 genes (14 positively and 28 negatively correlated), 42 genes (14 positively and 28 negatively correlated), 42 genes (14 positively and 28 negatively correlated) and 42 genes (28 positively and 14 negatively correlated), respectively (Table S7). A gene-metabolite network graph to further examine the relationship between genes and metabolites (r > 0.99) showed that phenylalanine levels were strongly associated with *novel.37619* (r = 0.9976), *Sspon.01G0047030-1T* (r = 0.9965), *Sspon.06G0030640-2D* (r = 0.9950), *novel.54281* (r = −0.9998), *Sspon.05G0007010-4D* (r = −0.9988), *Sspon.04G0008060-1A* (r = −0.9986) and *Sspon.04G0008060-2B* (r = −0.9986) (Figure 9 and Table S7). Cinnamate was not strongly associated with any genes, but tyrosine was strongly associated with *Sspon.01G0047030-1T* (r = 0.9949), *novel.21112* (r = 0.9932), *novel.37619* (r = 0.9927), *novel.5557* (r = −0.9976), *Sspon.04G0008060-1A* (r = −0.9970) and *Sspon.05G0007010-4D* (r = −0.9966) (Figure 9 and Table S7). Phenethylamine was only strongly associated with *novel.8291* (r = −0.9935) (Figure 9 and Table S7). The strong correlation between these genes and metabolites suggested substantial positive/negative regulatory effects under DS.

**Figure 10:**
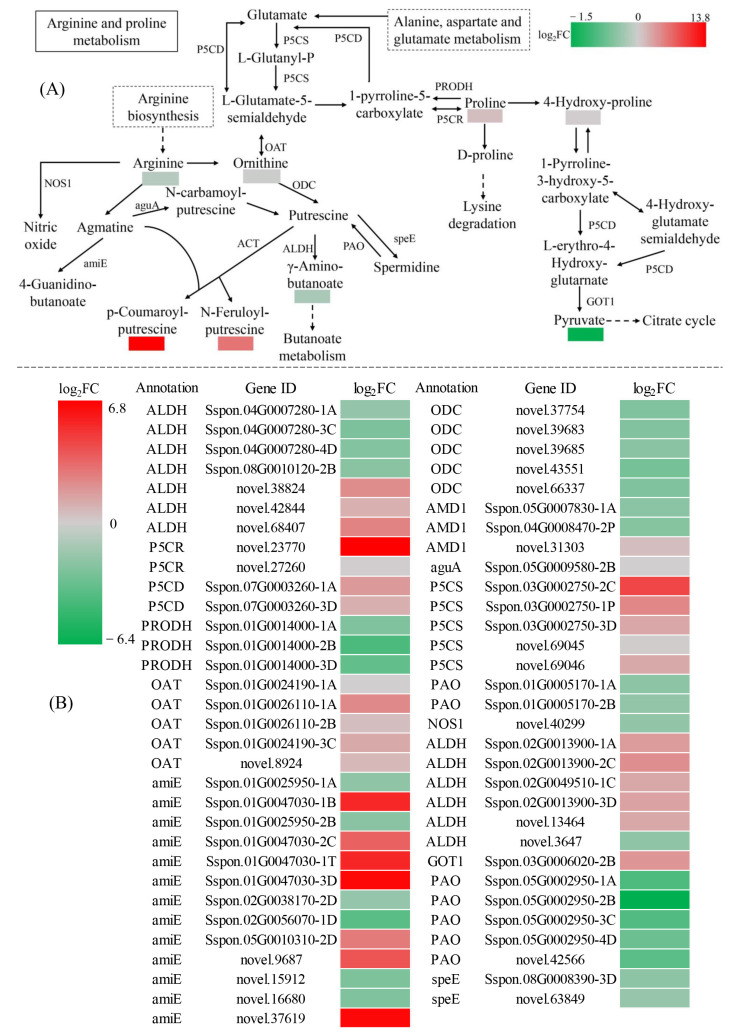
Analysis of DEGs and SRMs in the arginine and proline metabolism pathway. (**A**) Enriched SRMs for the CG/DS groups. (**B**) Enriched DEGs for the CG/DS groups. |log_2_FC| denotes |log_2_Fold Change|. Solid line stands for direct reaction. Dashed line stands for indirect reaction, which means it have to go through multiple reactions.

**Figure 11:**
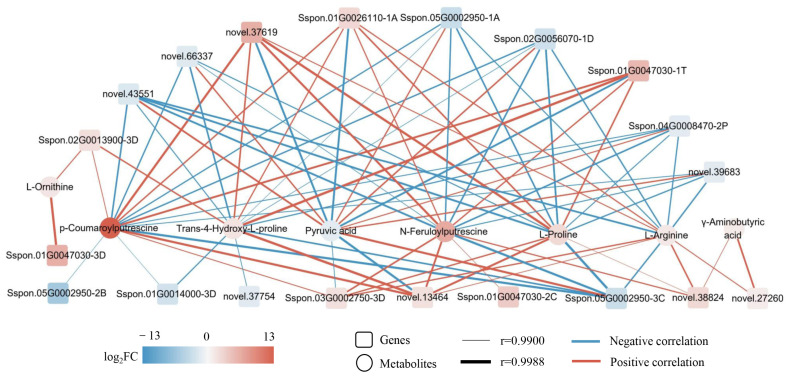
Correlation network graph of DEGs and SRMs in the arginine and proline metabolism pathway for comparison of the CG and DS groups. The genes and metabolites color-coded according to fold-change between the CG and DS groups. |log_2_FC| denotes |log_2_Fold Change|.

The Arginine and proline metabolism pathway had enrichment of eight SRMs (seven up-regulated and one down-regulated) and 63 DEGs (32 up-regulated and 31 down-regulated) (Figure 10 and Table S8). Up-regulated SRMs included proline, 4-hydroxy-proline, arginine, ornithine, γ-amino-butanoate (GABA), *p*-coumaroyl-putrescine and N-feruloyl-putrescine (Figure 10 and Table S8). Pyruvate was the only down-regulated metabolite. The *ODC* and *PAO* DEGs were down-regulated, and of the eight *ALDH* DEGs were up-regulated and five *ALDH* DEGs were down-regulated. These changes were accompanied by decreased amounts of putrescine and increases in GABA, spermidine and ornithine. Therefore, Arginine and proline metabolism responded positively to DS, resulting in synthesis of higher amounts of ornithine, arginine and proline that could contribute to improvements in drought tolerance. Meanwhile, reductions in pyruvate content are related to reduced respiration and overall slowing of growth activities in sugarcane. Gene-metabolite correlation analysis (r > 0.9, *p* < 0.01) for ornithine, arginine and proline showed significant correlation with 43 genes (23 positively and 20 negatively correlated), 38 genes (19 positively and 19 negatively correlated) and 43 genes (24 positively and 19 negatively correlated), respectively (Table S9). In particular, ornithine was strongly associated with *Sspon.01G0047030-3D* (r = 0.9984) and *Sspon.02G0013900-3D* (r = 0.9927) (Figure 11 and Table S9). Arginine was strongly associated with *novel.38824* (r = 0.9950), *Sspon.03G0002750-3D* (r = 0.9942), *Sspon.01G0026110-1A* (r = 0.9936), *novel.37619* (r = 0.9927), *novel.27260* (r = 0.9924), *novel.13464* (r = 0.9914), *novel.43551* (r = −0.9961), *novel.39683* (r = −0.9947), *Sspon.05G0002950-3C* (r = −0.9946), *Sspon.02G0056070-1D* (r = −0.9944), *Sspon.04G0008470-2P* (r = −0.9939) and *Sspon.05G0002950-1A* (r = −0.9931) (Figure 11 and Table S9). Proline was strongly associated with *novel.37619* (r = 0.9984), *novel.13464* (r = 0.9971), *Sspon.01G0026110-1A* (r = 0.9954), *Sspon.01G0047030-1T* (r = 0.9951), *Sspon.03G0002750-3D* (r = 0.9943), *novel.38824* (r = 0.9900), *Sspon.05G0002950-3C* (r = −0.9987), *novel.43551* (r = −0.9968), *Sspon.02G0056070-1D* (r = −0.9962), *Sspon.05G0002950-1A* (r = −0.9953), *novel.39683* (r = −0.9940), *novel.66337* (r = −0.9931) and *Sspon.04G0008470-2P* (r = −0.9927) (Figure 11 and Table S9). These genes and metabolites involved in Arginine and proline metabolism likely play an important role in sugarcane osmoregulatory responses to DS.

## 3. Discussion

### 3.1. Global Analysis of Phenotype, Physiology, Transcriptome and Metabolome Response to DS by Sugarcane

Drought is one of the most serious abiotic stresses and affects global crop production [16]. Sugarcane is most sensitive to DS during the tillering and elongation stages as evidenced by inhibited growth of stems and leaves [2,17]. In this study, we examined molecular regulation mechanisms of sugarcane in response to DS during the tillering stage. Responses to DS by sugarcane include leaf yellowing, leaf rolling, inhibition of stalk and leaf growth, and reduced biomass [18]. We observed similar phenotypes for Badila exposed to DS. Moreover, the chlorophyll content, photosynthesis (Photo), stomatal conductance (Cond), intercellular carbon dioxide concentration (Ci) and transpiration rate (Trmmol) were all markedly decreased under DS, showing that DS has significant negative effects on photosynthesis in sugarcane. During DS, there is less water available for absorption, and one response to limited water is a reduction in stomata size [19,20]. To adapt to DS, plants synthesize an array of substances that contribute to osmotic regulation including proline, glycine-betaine, carbohydrates and some inorganic ions [5,21]. Here, we observed that the proline content, soluble sugar content and malonaldehyde (MDA) content all increased significantly under DS, indicating that DS is also accompanied by osmotic stress and oxidative stress.

In addition to the abovementioned changes, the transcriptome changed in response to DS. Compared to the CG group with normal watering, plants in the DS group exhibited 13,744 DEGs (6663 up-regulated DEGs and 7081 down-regulated DEGs). The top five GO terms of biological process, cellular component and molecular function were related to photosynthesis, and the enriched DEGs had down-regulated expression. Moreover, the top five KEGG enriched pathways were carbon fixation in photosynthetic organisms, photosynthesis—antenna proteins, photosynthesis, carbon metabolism and starch and sucrose metabolism, and the enriched DEGs also had down-regulated expression. These results align with those for KPS01-12 sugarcane (drought-tolerant genotype) in a study by Nawae et al., which showed that expression levels of genes involved in water retention, oxidative and osmotic stress were higher than those for UT12 (drought-sensitive genotype), and that UT12 had more down-regulated genes involved in photosynthesis, carbon fixation and the Calvin cycle than KPS01-12 [22]. The results are also similar to a transcriptome analysis comparing maize having drought tolerant or susceptible genotypes that indicated high enrichment of DEGs in photosynthesis, histone, and carbon fixation pathways in the drought-susceptible line [23].

The significant negative effect of DS on photosynthesis in Badila at the transcriptome level was consistent with photosynthetic rate measurements. In general, plants respond to DS by reprogramming transcriptional, proteomic, and metabolic pathways to protect cells from stress-mediated damage [24,25]. Here, DS-induced changes in gene expression were associated with changes in metabolite levels. We identified 166 SRMs and most (129) were up- rather than down-regulated (37) in response to DS. In rice, Ham et al. also found that drought significantly enhanced levels of γ-aminobutyric acid (GABA, 244.6%), fructose (155.7%), glucose (211.0%), glycerol (57.2%), glycine (65.8%) and aminoethanol (192.4%) in transgenic grains compared to non-transgenic control grains [26]. Ma et al. hypothesized that the metabolites protected photosynthesis activity under DS via osmotic adjustments and/or antioxidant mechanisms [27]. Similarly, the SRMs identified in this study likely protect photosynthesis and growth activities of sugarcane. These SRMs were classified as alkaloids, amino acids and derivatives, lipids, flavonoids and phenolic acids that accounted for 19.28%, 18.67%, 14.46%, 14.46% and 13.25%, respectively, of the SRMs identified. As such, alkaloids and amino acids and their derivatives were the main SRMs affected by DS in sugarcane. In many plant species, accumulation of amino acids and their derivatives is induced by stress [11,28]. KEGG analysis showed that Aminoacyl-tRNA biosynthesis, 2-Oxocarboxylic acid metabolism, Biosynthesis of amino acids, Phenylalanine metabolism, and Arginine and proline metabolism were the most enriched in this study. Furthermore, combined transcriptome and metabolome analysis showed that sugarcane rind color was closely related to Flavonoid biosynthesis [29]. Cyanidin, cyanidin 6′-malonylglucoside, cyanidin O-glucoside and peonidin O-glucoside were the main flavonoid metabolites, and seven genes related to anthocyanin biosynthesis were candidate regulatory genes [29]. In combined transcriptome and metabolome analyses to compare control plants with plants exposed to DS (T_CG/DS and M_CG/DS, respectively), we found the same KEGG-enriched pathways (Biosynthesis of amino acids, Phenylalanine metabolism and Arginine and proline metabolism). These results highlight the potential importance of Phenylalanine metabolism and Arginine and proline metabolism as well as the usefulness of combining transcriptome and metabolome analyses for exploring important pathways for regulation of drought resistance.

### 3.2. Phenylalanine Metabolism in Sugarcane Is Affected by DS

Phenylalanine metabolism is an important secondary metabolic pathway in plants that directly or indirectly produces a variety of phenyl propanoid metabolites including phytoalexin, lignin, flavonoids and hydroxycinnamic acid amides [30]. Phenyl propanoid metabolites are closely related to plant growth and development and also play a vital role in responses to biotic and abiotic stresses [31]. In this study, we found that Phenylalanine metabolism actively participated in drought resistance regulation in sugarcane at a molecular level. Five up-regulated SRMs (phenylalanine, phenethylamine, N-acetyl-L-phenylalanine, 2-phenybacetamide and tyrosine) and two down-regulated SRMs (cinnamate and pyruvate) were enriched in the Phenylalanine metabolism pathway under DS. This change could enhance the antioxidant capacity of sugarcane. Pyruvate is a key intermediate metabolite that is important for photosynthesis and respiration [32]. With the decrease in pyruvate content, glycolysis activity also decreased, and plant growth slowed. Under DS, we found that 60 DEGs (17 up-regulated and 43 down-regulated) in the Phenylalanine metabolism pathway were enriched, including *hbd* (*3-hydroxybutyryl-CoA dehydrogenase*), *TAT* (*tyrosine aminotransferase*), *amiE* (*amidase*), *DDC* (*aromatic-L-amino-acid decarboxylase*), *echA* (*enoyl-CoA hydratase*), *PAL* (*phenylalanine ammonia-lyase*), *PTAL* (*phenylalanine/tyrosine ammonia-lyase*), *GOT1* (*aspartate aminotransferase 1*) and *AOC* (*primary-amine oxidase*). PAL is the first enzyme in the phenylpropanoid pathway and plays a vital role in plant growth as well as disease defense and resistance [33]. Multiple studies have shown that PAL activity is positively correlated with accumulation of lignin, anthocyanins, flavones and flavonoids, indicating that PAL activity plays an important role in regulating the generation of secondary metabolites [34]. Here, we identified 12 *PAL* DEGs that were down-regulated. Of these, *novel.31261*, *Sspon.04G0008060-1A*, *Sspon.04G0008060-2B* and *Sspon.04G0008060-3C* had the most significant correlation with seven SRMs. Therefore, these four PAL genes likely have significant regulatory effects on the phenylalanine metabolism pathway of sugarcane under DS and could be used as candidate genes for DS regulation.

### 3.3. Arginine and Proline Metabolism in Sugarcane Response to DS

Arginine and proline metabolism is critical not only for plant nitrogen assimilation, signal transduction and other physiological and biochemical processes, but also for osmotic regulation in plants under DS [35,36,37]. In maize, production of these two amino acids and their metabolites is induced by abiotic stress, as evidenced by the substantial accumulation of proline, arginine and γ-aminobutyrate in leaves [38]. Here, we obtained a similar result with the observation of upregulated of levels of proline, 4-hydroxy-proline, arginine, ornithine, γ-amino-butanoate (GABA), *p*-coumaroyl-putrescine and N-feruloyl-putrescine that are involved in the Arginine and proline metabolism pathway, indicating that these metabolites actively contribute to osmotic adjustments in sugarcane under DS. Several studies have shown that plants can rapidly accumulate large amounts of proline in response to abiotic stress [39]. Under stress conditions, proline serves as a compatibility osmotic regulator, protein and subcellular structure stabilizer, reactive oxygen scavenger and redox equilibrator [40]. In wheat, expression of *P5CS* and *P5CR* was previously shown to be up-regulated and *PRODH* was down-regulated in response to salinity stress [41]. We found similar results in this study. DEGs related to proline synthesis (*P5CS*, *P5CD*, *P5CR* and *OAT*) were up-regulated, and *PRODH*, which is involved in proline degradation, was down-regulated. Furthermore, gene-metabolite correlation analysis (r > 0.9, *p* < 0.01) showed that proline levels had a significant positive correlation with expression of *P5CS*, *P5CD*, *P5CR* and *OAT* genes, and a significant negative correlation with *PRODH*. The gene-metabolite correlation network graph analysis (r > 0.99) showed that proline was strongly associated with *Sspon.01G0026110-1A* (*OAT*) and *Sspon.03G0002750-3D* (*P5CS*). Together, these results suggest that *Sspon.01G0026110-1A* and *Sspon.03G0002750-3D* are related to proline synthesis and could be candidate genes for enhancing drought tolerance in sugarcane.

## 4. Materials and Methods

### 4.1. Plant Materials and Experimental Design

The drought-sensitive cultivar Badila (*Saccharum officinarum*) used in this study was provided by the National Engineering Research Center for Sugarcane, Fujian Agriculture and Forestry University. Sugarcane stems were cut into single-bud segments that were soaked in 0.5% carbendazim solution for 24 h before placing in pots (12 cm diameter and height) containing pine needle soil. The pots were kept on an open balcony (23–28 °C). When the seedlings reached the 2-leaf stage, 3 seedlings for each treatment with consistent growth potential were transferred to a bucket (45 cm diameter, 30 cm high) with 35 kg mixed soil (clay soil:pine needle soil = 3:1 (*v*:*v*)). Six buckets, labeled 1–6, were placed in a greenhouse and watered every 2 days. When the plants reached the 8-leaf stage, plants in buckets 1–3 (drought stress, DS) received no additional watering, whereas those in buckets 4–6 (control group, CG) were watered as usual.

### 4.2. Measurement of Physiological Parameters

At 9:00 pm on day 10 of exposure to DS, the photosynthetic activity of the leaves was measured with a portable photosynthesis system (LI6400, Lincoln, NE, USA) and the leaf chlorophyll content was measured with a chlorophyll meter (SPAD-502PLUS, Tokyo, Japan). The +1 and −1 leaves of plants in each group were clipped, mixed and placed in a Ziploc bag before freezing in liquid nitrogen. These samples were used for physiological experiments, RNA sequencing and metabolome analyses. Free proline and soluble sugar concentrations were determined using the acid ninhydrin reagent method and anthrone method, respectively [42]. Malondialdehyde (MDA) concentration was determined using the thiobarbituric acid method [43].

### 4.3. RNA Sequencing and Data Analysis

Six samples of total RNA were extracted using NA Isolater Total RNA Extraction Reagent according to the manufacturer’s instructions (Novizan Biotechnology Co., Ltd., Nanjing, China). The samples were termed T_CG (control group) and T_DS (drought stress). The cDNA library was constructed and sequenced on an Illumina HiSeq X-ten platform at the Wuhan Metware Biotechnology Co., Ltd. (Wuhan, China) using 150 bp paired end reads (PE 150). To obtain clear reads, low-quality reads, adapter sequences and sequences having >10% poly-N in the raw reads were filtered. The Q20, Q30 and GC contents of each sample were calculated. Clear reads from every sample were mapped to the *Saccharum spontaneum* [44] reference genome using HISAT2 [45]. Raw counts of genes were determined using feature counts [46]. Differentially expressed genes (DEGs) between two samples were identified using DESeq2 with |log_2_fold change| ≥ 1 and a false discovery rate (FDR) < 0.05 [47]. DEG function was annotated using the KEGG and GO databases. KEGG pathway analysis of DEGs was performed with BLAST software [48] and KEGG enrichment was analyzed using KOBAS 2.0 software with *p*-value < 0.05 [49,50]. GO analysis of DEGs was carried out using the R package clusterProfiler [51]. Transcription factors (TFs) among the DEGs were predicted using iTAK [52] software with PlnTFDB [53] and PlantTFDB [54] databases.

### 4.4. Real-Time Quantitative PCR (RT-qPCR)

Twelve DEGs belonging to the Phenylalanine metabolism and Arginine and proline metabolism pathways were verified by RT-qPCR to evaluate the accuracy of RNA-Seq. Based on the coding gene sequences, RT-qPCR primers were designed using primer premier 6.0 software (PREMIER Biosoft, San Francisco, CA, USA). Glyceraldehyde-3-phosphate dehydrogenase (*GAPDH*) was selected as the internal control gene (Table S2). SYBR^®^ Green Premix Pro Taq HS qPCR Kit (Novizan Biotechnology Co., LTD, Nanjing, China) was used for the RT-qPCR assay using 20 μL reaction solutions containing 10 μL 2 × ChamQ Universal SYBR qPCR Master Mix, 0.5 μL primer F (10 μM), 0.5 μL primer R (10 μM), 1 μL cDNA and 8 μL nuclease-free water. The qPCR reactions involved denaturation at 95 °C for 30 s, followed by 40 cycles of 5 s at 95 °C and 30 s at 60 °C. The RT-qPCR assays were carried out using a QuantStudio^®^ Real-Time PCR system (Applied Biosystems, Foster City, CA, USA). The qPCR data were analyzed using the 2^−ΔΔCt^ quantitative method to determine differences in gene expression [55]. Three independent biological replicates and three technological replicates were used for each sample in this study.

### 4.5. Widely Targeted Metabolomics

For metabolomic analyses, three biological replicates for each group termed M_CG (control group) and M_DS (drought stress group) were used. Sample preparation, extraction, identification, and quantification of the widely targeted metabolome was performed by Wuhan Metware Biotechnology Co., Ltd. (Wuhan, China). Biological samples were freeze-dried in a vacuum freeze-dryer (Scientz-100F, Ningbo, China) and then crushed using a mixer mill (MM 400, Retsch, Shanghai, China) with a zirconia bead for 1.5 min at 30 Hz. Then, 100 mg of lyophilized powder was dissolved in 1.2mL 70% methanol solution, vortexed for 30 s every 30 min for a total of 6 times, and incubated at 4 °C overnight. Following centrifugation (ThermoFisher, Osterode, Germany) at 12,000 rpm for 10 min, the extracts were filtered (SCAA-104, 0.22μm pore size; ANPEL, Shanghai, China) before use in UPLC-MS/MS analysis.

### 4.6. UPLC Conditions

The sample extracts were analyzed using a UPLC-ESI-MS/MS system (UPLC, SHIMADZU Nexera X2, Kyoto, Japan; MS, Applied Biosystems 4500 Q TRAP, Shanghai, China). An Agilent SB-C18 (1.8 µm, 2.1 mm × 100 mm) column was used. The mobile phase consisted of solvent A, pure water with 0.1% formic acid, and solvent B, acetonitrile with 0.1% formic acid. Sample measurements were performed with a gradient program beginning with 95% A, 5% B that reached 5% A, 95% B within 9 min via a linear gradient. The 5% A, 95% B condition was maintained for 1 min. Subsequently, a composition of 95% A, 5% B was reached within 1.1 min and maintained for 2.9 min. The flow rate was 0.35 mL per minute. The column oven was set to 40 °C and the injection volume was 4 μL. The effluent was alternatively connected to an ESI-triple quadrupole-linear ion trap (QTRAP)-MS.

### 4.7. ESI-Q TRAP-MS/MS

LIT and triple quadrupole (QQQ) scans were acquired on a triple quadrupole-linear ion trap mass spectrometer (Q TRAP), AB4500 Q TRAP UPLC/MS/MS System, equipped with an ESI Turbo Ion-Spray interface, operating in positive and negative ion mode and controlled by Analyst 1.6.3 software (AB Sciex, Framingham, MA, USA). The ESI source operation parameters were: ion source, turbo spray; source temperature 550 °C; ion spray voltage (IS) 5500 V (positive ion mode)/-4500 V (negative ion mode); ion source gas I (GSI), gas II(GSII), and curtain gas (CUR) were set at 50, 60 and 25.0 psi, respectively; the collision-activated dissociation (CAD) was high. Instrument tuning and mass calibration were performed with 10 and 100 μmol/L polypropylene glycol solutions in QQQ and LIT modes, respectively. QQQ scans were acquired as MRM experiments with collision gas (nitrogen) set to medium. DP and CE for individual MRM transitions were conducted with further DP and CE optimization. A specific set of MRM transitions was monitored for each period according to the metabolites eluted within the period.

### 4.8. Metabolome Data Analysis

Results for hierarchical cluster analysis (HCA) of samples and metabolites are presented as heatmaps with dendrograms. Pearson correlation coefficients (PCC) between samples were calculated using the cor function in R (accessed on: 10 December 2021) and presented as only heatmaps. Both HCA and PCC were carried out using the R package pheatmap. For HCA, normalized signal intensities of metabolites (unit variance scaling) are visualized as a color spectrum. Principal component analysis (PCA) was performed with the statistics function prcomp within R. The data were unit variance scaled before PCA. Significantly regulated metabolites (SRMs) between groups were determined by VIP ≥ 1 and absolute Log_2_FC (fold change) ≥ 1. VIP values were extracted from OPLS-DA results, which also contained score plots and permutation plots, and was generated using R package MetaboAnalystR. The data were log transformed (log_2_) and mean centered before OPLS-DA. To avoid overfitting, a permutation test (200 permutations) was performed. Identified metabolites were annotated using the KEGG compound database (accessed on: 10 December 2021) and annotated metabolites were then mapped to the KEGG pathway database (accessed on: 10 December 2021). Pathways with the SDMs mapped were subjected to MSEA (metabolite sets enrichment analysis), and their significance was determined using *p*-values from a hypergeometric test.

### 4.9. Statistical Analysis

Duncan’s multiple comparison method was conducted using Statistical Product and Service Solutions software (IBM SPSS 19.0, 2010, North Castle, NY, USA) to assess differences in physiological indices across three different samples for each treatment (*p* < 0.05). The bar diagram was drawn using Microsoft Office Excel 2016 (Microsoft, Redmond, WA, USA). Pearson correlation analysis of gene-metabolites was performed using SPSS 19.0 (threshold for association analysis > 0.9, *p* < 0.01). The gene-metabolite correlation network diagram was visualized using Cytoscape 3.8.2 [56].

## 5. Conclusions

In this study, drought stress (DS) significantly affected sugarcane growth. Physiological results indicated that photosynthesis activity decreased significantly under DS. Moreover, transcriptome GO and KEGG enrichment analysis showed that differentially expressed genes (DEGs) under DS were mainly enriched in metabolic pathways related to photosynthesis, and most of the DEGs were down-regulated. Therefore, the adverse effect of DS on photosynthesis is an important factor that slows the growth of sugarcane plants. DS also significantly affected synthesis and accumulation of metabolites. Most of the identified SRMs were up-regulated in response to DS and over 50% of SRMs were alkaloids, amino acids and their derivatives, and lipids. Combined transcriptome and metabolome analysis showed that Biosynthesis of amino acids, Phenylalanine metabolism, and Arginine and proline metabolism were common KEGG enriched pathways in both T_CG/DS and M_CG/DS. Further analyses indicated that Phenylalanine metabolism and Arginine and proline metabolism pathways were closely related to regulation of drought tolerance in sugarcane. These results highlight the potential importance of Phenylalanine metabolism and Arginine and proline metabolism, of which genes and metabolites in two metabolism pathways will play a critical role to improve sugarcane drought tolerance.

## Data Availability

The raw read data of RNA-seq have been deposited in the GSA (accession: CRA007750), China National Center for Bioinformationer/Beijing Institute of Genomics, Chinese Academy of Sciences (https://ngdc.cncb.ac.cn/gsa/, accessed on 10 December 2021, Beijing, China). The metabolome data reported in this paper have been deposited in the OMIX (accession: OMIX001540), China National Center for Bioinformation/Beijing Institute of Genomics, Chinese Academy of Sciences (https://ngdc.cncb.ac.cn/omix/, accessed on 10 December 2021, Beijing, China).

## Supplementary Materials

The following supporting information can be downloaded at: https://www.mdpi.com/article/10.3390/ijms24043856/s1.
