# Supplementary material for: Global Responses of Autopolyploid Sugarcane Badila (Saccharum officinarum L.) to Drought Stress Based on Comparative Transcriptome and Metabolome Profiling
Source: Int J Mol Sci. 2023 Feb 14;24(4):3856. doi: 10.3390/ijms24043856 (PMC9966050; doi:10.3390/ijms24043856)
Supplement: Supplementary file 1 [file ijms-24-03856-s001.zip › ijms-2111851 Supplementary Figures.pdf]

Global Responses of Autopolyploid Sugarcane Badila (*Saccharum officinarum* L.) to Drought Stress Based on Comparative Transcriptome and Metabolome Profiling

Shan Yang [1], Na Chu [2], Naijie Feng [1], Bolin Zhou [1], Hongkai Zhou [1], Zuhu Deng [2], Xuefeng Shen [1,*] and Dianfeng Zheng [1,*]

1 College of Coastal Agricultural Sciences, Guangdong Ocean University, Zhanjiang 524088, China

2 National Engineering Research Center for Sugarcane, Fujian Agriculture and Forestry University, Fuzhou 350002, China

* Correspondence: shenxuefeng@gdou.edu.cn (X.S.); zhengdf@gdou.edu.cn (D.Z.

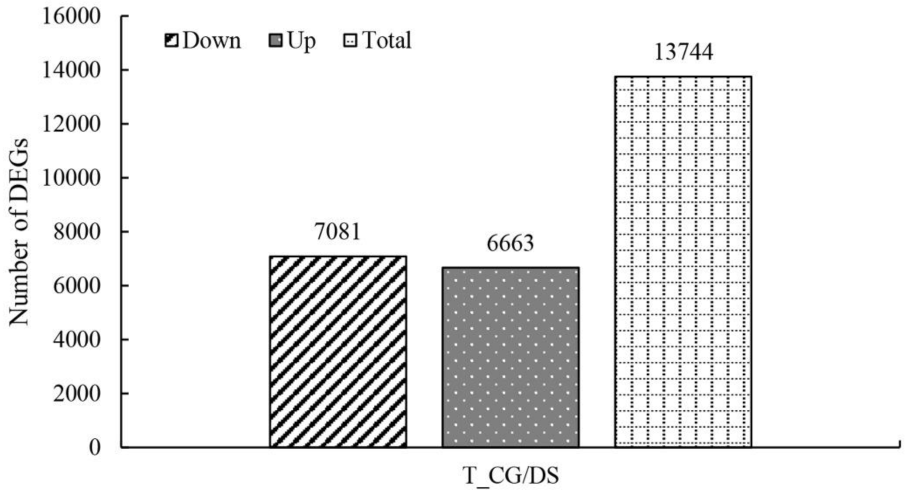

Figure S1. Number of DEGs between the CG and DS groups. The total number of DEGs is represented by the bar on the right and down- and up-regulated genes are represented by the bars on the left and center, respectively.

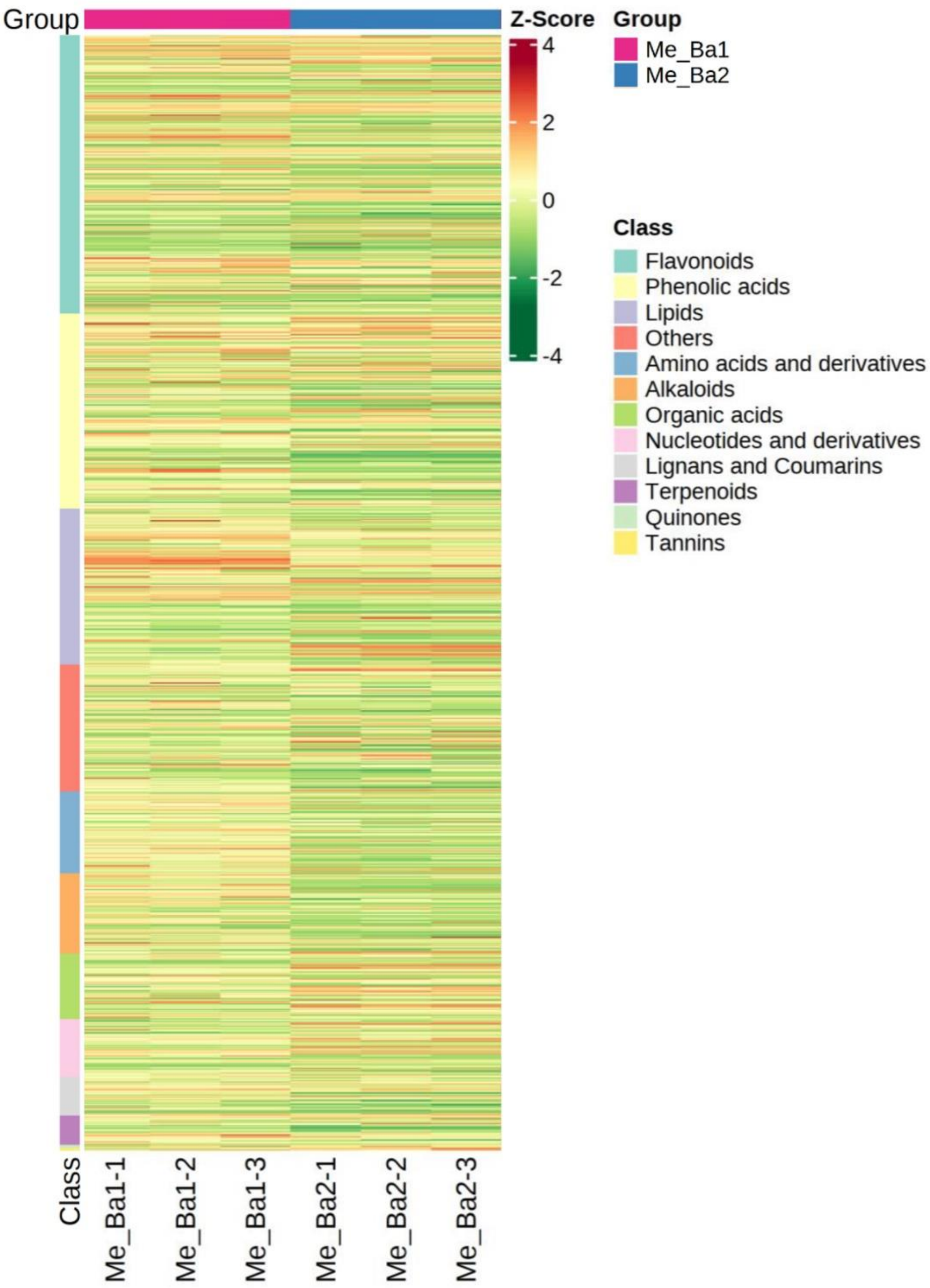

Figure S2. Heatmap hierarchical clustering of all detected metabolites. Me_Ba1 (pink, left column) and Me_Ba2 (blue, right column) represent the metabolome for three different DS and CG plants, respectively. Gene classes are arrayed in rows and color coded according to the key.

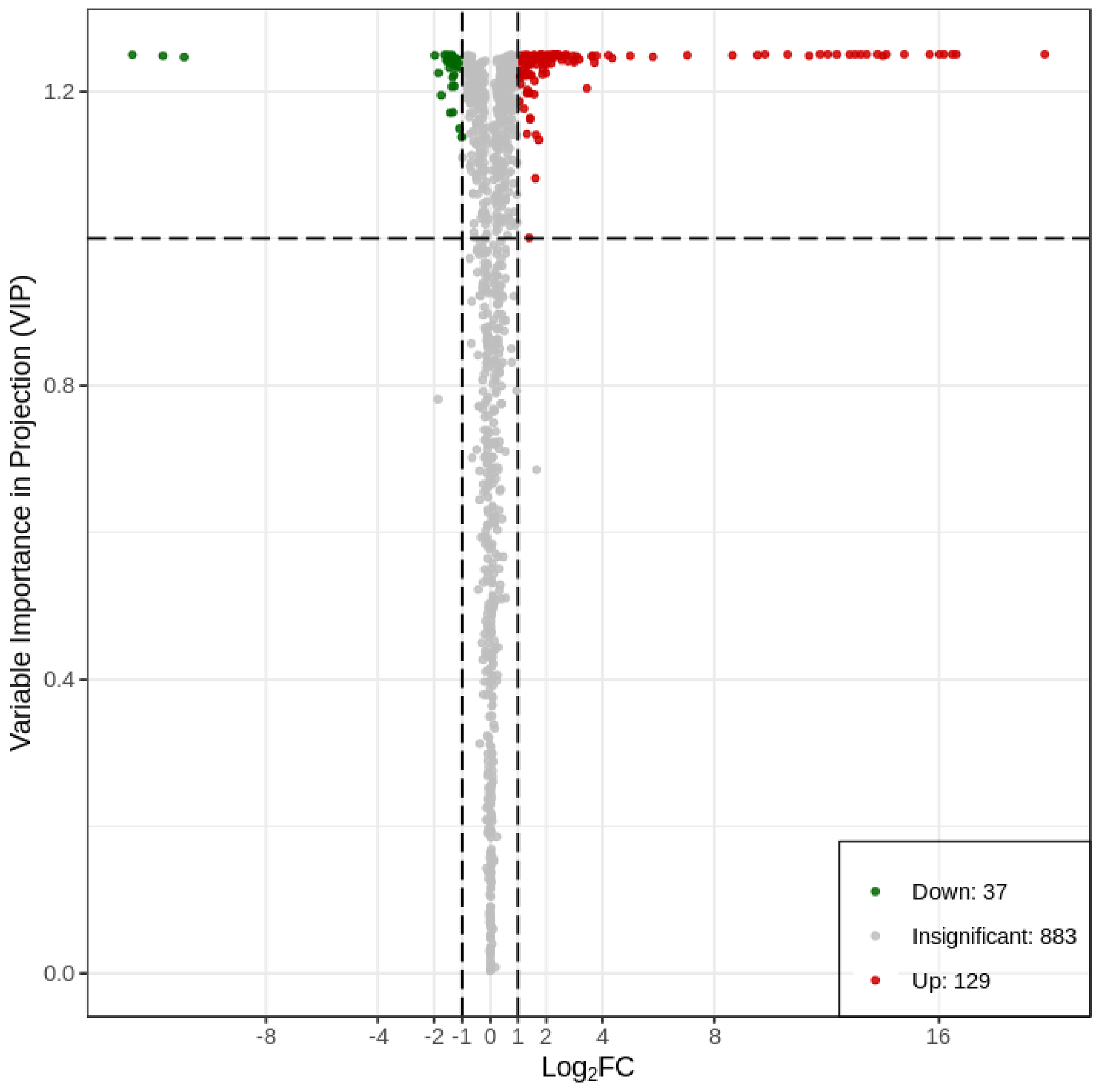

Figure S3. Volcano plot of significantly regulated metabolites (SRMs) based on fold-change between the CG and DS groups.

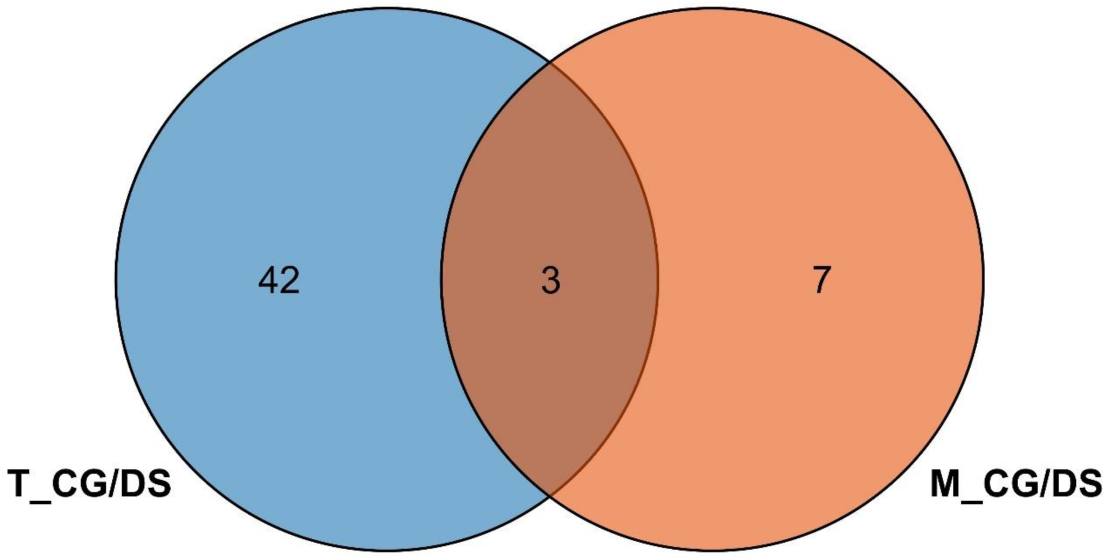

Figure S4. Venn diagram of the comparison between transcriptome (T_CG/DS) and metabolome (M_CG/DS) for CG and DS plants.
